# Evaluation of Perceptual Realism and Clinical Plausibility of AI-Generated Colon Polyp Images

**DOI:** 10.3390/biomedicines13071561

**Published:** 2025-06-26

**Authors:** Andrei-Constantin Ioanovici, Andrei-Marian Feier, Marius-Ștefan Mărușteri, Vasile Florin Popescu, Daniela-Ecaterina Dobru

**Affiliations:** 1 Department M2—Complementary Functional Sciences, Medical Informatics and Biostatistics, George Emil Palade University of Medicine, Pharmacy, Science, and Technology of Targu Mures, 540142 Targu Mures, Romania; andrei.ioanovici@umfst.ro; 2 Department M4—Clinical Sciences, Orthopedics and Traumatology I, George Emil Palade University of Medicine, Pharmacy, Science, and Technology of Targu Mures, 540139 Targu Mures, Romania; andrei.feier@umfst.ro; 3 Information Systems and Cyber Defense Department, “Carol I” National Defense University, Sos. Panduri no. 68-72, 050662 Bucharest, Romania; popescuveve@gmail.com; 4 Department M4—Clinical Sciences, Gastroenterology Medical VII, George Emil Palade University of Medicine, Pharmacy, Science, and Technology of Targu Mures, 540139 Targu Mures, Romania; daniela.dobru@umfst.ro

**Keywords:** colon polyps, synthetic data, pseudosynthetic data, colonoscopy

## Abstract

**Background:** Synthetic and pseudosynthetic images can be used to extend colonoscopy datasets, which, in turn, are used to train AI-detection models, yet their clinical acceptability depends on whether medical professionals can still recognize non-real content. **Aim:** To quantify the ability of practicing gastroenterologists to discriminate real, pseudosynthetic, and synthetic polyp images and to determine how training level and synthesis method impact detection. **Materials and Methods:** A total of 32 Romanian gastroenterologists (18 residents and 14 seniors) reviewed 24 images (8 real, 8 augmented, 4 CycleGAN, and 4 diffusion) via an online form. Classification accuracy, 95% confidence intervals (CI), class sensitivity and precision, 3 × 3 confusion matrices, and Fleiss’ κ were calculated. Resident vs. senior differences were tested with Pearson χ^2^; CycleGAN versus diffusion detectability was analyzed with the Wilcoxon signed-rank test (α = 0.05). **Results:** Overall accuracy was 61.2% (95% CI 57.7–64.6). Residents and seniors performed similarly (62.3% vs. 59.8%; χ^2^_1_ = 0.38, *p* = 0.54). Sensitivity/precision were 70.7%/62.2% for real, 51.6%/58.9% for augmented, and 61.3%/62.1% for synthetic images. Collapsing to “real vs. non-real” yielded 70.7% sensitivity and 78.5% specificity for real images. CycleGAN images were always recognized as synthetic (128/128; 97.1–100% CI), whereas diffusion images were correctly classified only 22.7% of the time (16.3–30.6%; Wilcoxon *p* < 0.001). The training level did not impact detection performance (χ^2^_2_ < 1.2, *p* > 0.5). Inter-rater agreement was fair (κ = 0.30, 95% CI 0.15–0.43). **Conclusions:** Clinicians detect non-real colonoscopy images only slightly above chance, irrespective of experience. The diffusion synthesis method creates images that escape human scrutiny, suggesting the need for automated authenticity safeguards before synthetic datasets are applied in clinical or AI-validation contexts.

## 1. Introduction

Colorectal cancer (CRC) remains one of the leading causes of cancer-related morbidity and mortality worldwide, with early detection and removal of precancerous polyps being the most effective method of reducing incidence and improving outcomes [1,2,3,4]. While colonoscopy is the gold standard for detection, even experienced endoscopists are dealing with substantial adenoma miss rates (AMRs), which can approach 26% based on a 2019 meta-analysis [5]. Recent advances in artificial intelligence (AI), particularly in deep learning-based image segmentation, offer a promising solution to enhancing diagnostic accuracy by supporting clinicians during endoscopic procedures [6,7,8,9].

Deep convolutional neural networks (CNNs) trained on large, diverse datasets have shown encouraging results in polyp detection and segmentation tasks. However, the development of such models is restricted by a bottleneck: the scarcity of annotated medical imaging data. Regulatory, ethical, and logistical constraints often limit access to sufficiently diverse datasets [10,11]. To address this challenge, two complementary approaches have gained attention: the generation of (1) synthetic data created from scratch using generative adversarial networks (GANs) or diffusion models [12,13,14] and (2) pseudosynthetic data, which consist of real images augmented via clinically plausible transformations (e.g., flipping, rotation, and contrast adjustment) while preserving a traceable link to the original case [14].

Our prior research has shown that training CNNs on datasets enriched with synthetic and pseudosynthetic images leads to significant performance gains in polyp segmentation models [15]. Diffusion-based synthetic images have been observed to offer greater generalization and anatomical realism than those generated via GANs. Meanwhile, pseudosynthetic data offer an advantage—traceability. Because these images are derived from real-world clinical cases, they maintain a direct connection to actual patient data, addressing concerns around privacy, reproducibility, and regulatory approval.

Despite these technical advances, an important aspect remains underexplored: Can human clinicians reliably distinguish between real, synthetic, and pseudosynthetic images? The clinical acceptability of AI-generated data depends not only on algorithmic performance but also on the trust and interpretive ability of its end users—gastroenterologists. If synthetic or pseudosynthetic images are perceptually indistinguishable from real ones, they may serve not only as valid training inputs for AI but also as educational tools and validation resources in medical practice.

In this study, we present a validation experiment designed to investigate gastroenterologists’ ability to discern between real, synthetic (CycleGAN and diffusion-generated), and pseudosynthetic images of colorectal polyps. While prior work demonstrated clear model performance benefits from using these augmented datasets, this work is investigating the perceptual realism of these images from a human point of view. By evaluating classification accuracy, confusion patterns, and differences between experience levels, we aim to assess the feasibility of integrating synthetic and pseudosynthetic datasets into clinical workflows and future doctor training platforms.

## 2. Materials and Methods

This study involved 32 gastroenterologists from university hospitals, state/public hospitals with no affiliation to a university, and private practices in Romania. The cohort included 14 senior gastroenterologists with an average of 6.1 years of post-residency experience and 18 residents with an average of 2.9 years of gastroenterology training. Inclusion criteria required active endoscopic practice. Participants were invited to fill out a questionnaire on Google Forms (Google LLC, Moutain View, CA, USA) via social media platforms and email and consented voluntarily [16]. Responses were anonymous, with no personal data collected. This study used publicly available, deidentified datasets.

A total of 24 endoscopic polyp images were used, divided equally into three classification categories: real, pseudosynthetic, and synthetic (8 images each). All images underwent uniform preprocessing—resized to 256 × 256 pixels and normalized to a [0, 1] pixel value range to ensure consistent display. Real images were randomly selected from the Kvasir-SEG [17] and PolypGen [18] datasets, containing 1000 and 3762 annotated polyp images, respectively. These represented endoscopic images with no visual modifications beyond preprocessing. Pseudosynthetic images were derived from Kvasir-SEG and PolypGen, with additional transformations applied using Python’s (version 3.11) Albumentations library [19]. Transformations included spatial alterations (horizontal flips, 90-degree rotations, and 10–20% crops) and color adjustments (10–20% changes in brightness, contrast, and saturation). Images were randomly selected from a pool of augmented images to include varied transformations. Synthetic Images were extracted from the Synth-Colon dataset, generated using CycleGAN [20], and from a dataset using the diffusion-based polyp synthesis method (Polyp-DDPM [21,22]). Four images were randomly selected from each dataset to balance generation methods.

Overall accuracy was calculated for the entire cohort and separately for residents and seniors; Wilson 95% confidence intervals were reported [19]. Pearson χ^2^ test assessed resident versus senior accuracy [23]. Sensitivity (recall) and precision (positive predictive value or PPV) were computed for each true class, and a binary analysis (“real” versus “synthetic/pseudosynthetic”) supplied sensitivity and specificity for recognizing real images. Confusion matrices of size 3 × 3 were produced for the complete set of ratings and for each training-level subgroup [24,25,26]. We used a Wilcoxon signed-rank test to assess the within-rater difference to compare how clinicians recognized the two polyp synthesis methods [27,28]. Within each true class, resident-versus-senior predicted-label distributions were compared with 2 × 3 χ^2^ tests. Inter-rater agreement was measured with Fleiss’ κ for all raters and for each subgroup with corresponding 95% confidence intervals [29]. The significance threshold—alpha—was set at 0.05. Statistical analysis was performed using Python version 3.11 with SciPy version 1.9.3 and Pandas version 1.5.3 libraries, using Google Colab as the environment [30,31].

## 3. Results

**Figure 1:**
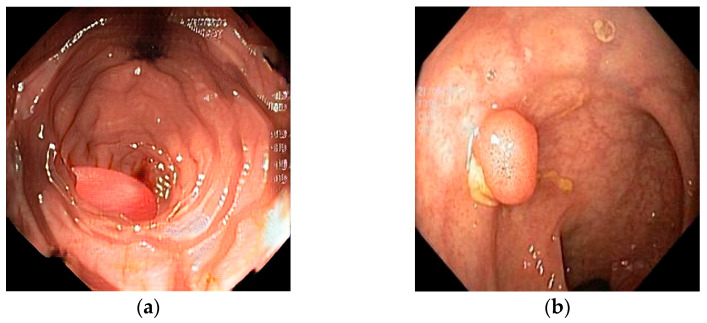
(**a**) Synthetic image generated using cGAN; (**b**) synthetic image generated using diffusion method.

For a better understanding of this study, two polar-opposite images are shown below that represent both ends of the perceptual–realism spectrum of synthetic generation. Figure 1 depicts a cGAN synthetic image (a), 100% reported by respondents as synthetic, next to a diffusion-based synthetic image (b), which was mislabeled by 26 out of 32 respondents (81.3%). The cGAN-generated image has color patterns and shapes that are easily recognized as synthetic, with the polyp having a “perfect” shape and the mucosa showing unnatural architecture. On the other hand, the diffusion-based image can be easily confused as real, displaying cues such as unnatural lighting and debris and mucosal folds with distortions. These features are not present in the real and augmented images. The real image that was mislabeled most frequently as synthetic or pseudosynthetic (12/32 or 37.5% contained mucosa with a normal vascular pattern with no sudden breaks, as well as normal colors and lighting. It contains a small, sessile colon polyp, showing the fine vascular detail and true texture that sometimes diffusion and almost always GAN models struggle to replicate.

Out of 768 ratings, 470 were correct, giving an overall accuracy of 61.2% (95% CI 57.7–64.6%). Residents provided 432 ratings and were correct on 269 occasions, for an accuracy of 62.3% (95% CI 57.6–66.7%). Seniors gave 336 ratings with 201 correct classifications, corresponding to an accuracy of 59.8% (95% CI 54.5–64.9%). Results are displayed in Table 1.

Pearson χ^2^ test shows no statistically significant difference in overall accuracy between residents and senior gastroenterologists (χ^2^) *p* = 0.54.

Across all 768 ratings, performance varied by image category. Real polyps were identified most consistently, while synthetic images showed a comparable precision but a slightly lower sensitivity, indicating that nearly four in ten computer-generated frames were mislabeled as either real or augmented. Augmented frames were the least recognizable. Performance metrics are reported in Table 2.

When the task was collapsed to a binary decision—“real” versus “synthetic/pseudosynthetic”—participants correctly detected genuine endoscopy frames with a sensitivity of 70.7% (181/256). They marked synthetic or pseudosynthetic images as non-real in 78.5% of cases (402/512).

We then computed the overall confusion matrix, with subsequent calculation of confusion matrices per each subset of participants (residents vs. seniors), and the results are displayed below in Table 3, Table 4 and Table 5. Performance is reported in percentages, along with 95% CI.

For each participant, the four CycleGAN images were paired with four diffusion images, creating 128 rater-level comparisons. Correct recognition (labeled synthetic) was perfect for CycleGAN (128/128) but achieved in only 29/128 cases for diffusion images.

Detection rates and 95% confidence intervals for each synthesis method are provided in Table 6.

We then applied the Wilcoxon signed-rank test, which returned a *p*-value of <0.001, confirming that CycleGAN frames were markedly easier to identify as synthetic.

The results regarding prediction patterns of residents vs. seniors within each true class are displayed in Table 7 below. None of the 2 × 3 comparisons reached statistical significance, indicating that residents and seniors distributed their response categories similarly for real, augmented, and synthetic images.

Illustrative cell counts and row percentages with 95% CIs are represented in Table 8, Table 9 and Table 10.

Across all three true classes, the 95% confidence intervals overlap by an appreciable degree, corroborating the statistically non-significant *p*-values.

Inter-rater agreement for the three-way task was modest. Fleiss κ for the entire group of 32 gastroenterologists was 0.30 (95% CI 0.15–0.43), a level conventionally interpreted as “fair” concordance. Agreement within the resident subgroup was slightly higher, κ = 0.34 (0.18–0.47), whereas seniors reached κ = 0.27 (0.11–0.40); the overlapping confidence intervals indicate no meaningful difference between experience levels.

## 4. Discussion

In this study, we saw that experience level did not materially shift the distribution of chosen labels, whereas the near-perfect recognition of CycleGAN output indicates obvious artifacts or texture cues that clinicians find easy to spot, whereas diffusion-generated images are far more realistic: more than three-quarters were mistaken for real or augmented. This disparity underscores that diffusion models currently pose the greater challenge for human verification in endoscopic workflows.

In this study, gastroenterologists achieved a moderate overall accuracy when distinguishing real, augmented, and synthetic polyp images, being in the same range as other findings in related domains, where observers usually score in the 55–65% range when tasked with real-vs-non-real image classification in endoscopy, radiology, and dermatology. For example, Yoon et al. conducted a study where four expert endoscopists evaluated GAN-synthesized colonoscopy images containing sessile serrated lesions. The experts’ ability to differentiate between real and synthetic images was limited, highlighting the challenges faced by clinicians in identifying AI-generated content.

Even with capsule endoscopy frames, gastroenterologists as a group performed near chance level (~53% correct) in differentiating real vs. GAN-generated images [32]. Similarly, radiologists reviewing synthetic versus real scans hover just above chance: for instance, a multi-reader study on high-resolution CT images reported a mean accuracy of 59.4%, only marginally better than the 50% expected by guessing [33]. In a Turing-style test with melanoma and nevus photos, both real and generated using GAN, 19 dermatologists (seniors and residents) achieved an overall accuracy of 59.3%. These findings show that overall accuracy in human realism discrimination falls in the 55–60% range for most modalities, indicating a substantial challenge in recognizing synthetic or augmented medical images [34].

Stratifying by experience level revealed no statistically significant difference in performance, suggesting that additional years of post-training endoscopic practice do not confer a clear advantage in perceiving subtle artifacts or anomalies introduced by modern augmentation and synthesis methods. One intuitive hypothesis is that having more years of experience might better detect subtle signs of image altering or synthesis, but studies show minimal benefit of observer experience regarding the type of task. In a CT study, radiologists were stratified by experience (junior residents, senior radiologists, and experts), but their ability to discern real vs. non-real images did not significantly differ (mean accuracies of 58.0%, 60.5%, and 59.8%, respectively; *p* = 0.36) [33].

There are cases where domain experts perform worse than their less-specialized colleagues. In a brain MRI experiment, the two fellowship-trained neuroradiologists achieved only 30% and 55% accuracy, essentially at or below chance, whereas three non-specialist radiologists scored between 64% and 83% [35]. Overall, the evidence indicates that observer background has a minor influence on detecting synthetic images, and expertise alone cannot overcome the fundamental visual plausibility of modern-era synthetic or augmented medical images.

When the classification experiment is collapsed to a binary (real-vs-non-real) decision, performance corresponds to an area under the curve in the 0.5–0.6 range—far below what would be needed for confident medical discrimination. Moreover, different people often disagree on which images are non-real, as reflected by poor inter-rater κ values in our study and the aforementioned studies.

These findings suggest the need for objective validation of image authenticity (for instance, algorithmic detection methods or metadata checks) if distinguishing real vs. AI-generated images is required, given that human observers alone are inclined to error and disagreement in this domain. The current literature provides a cautionary note: in the context of medical imaging, synthetic data generation techniques have effectively passed the “visual Turing test” in many cases, achieving deception rates that make human recognition unreliable [32,33,34,35].

The results in this research are in line with other published papers that demonstrate the superiority of diffusion methods over GAN methods in terms of generalization ability and anatomical authenticity. For example, Balla et al. (2023) found that a diffusion model produced synthetic ultrasound images whose pixel intensity distributions closely matched real images, preserving realistic anatomical textures, compared to GAN methods [36]. Additionally, the work of Saragih et al. from 2024 and Hung et al. (2023) have made similar comparisons between synthetic medical data generation methods and concluded that diffusion methods outperform GANs and the former have great potential regarding conditional medical image generation [37,38].

The design of this study is in line with other published papers, such as the work of Korkinof et al. from 2020, which asked participants to judge 10 image pairs (20 images) per reader when assessing GAN-derived mammograms [39]. We nevertheless acknowledge that broader lesion diversity could further strengthen generalizability, and future work will imply the expansion of an experiment with images from multiple medical institutions and potentially incorporate video sequences, where temporal cues might aid authenticity judgments, better replicating the endoscopy process. Because our cohort consisted only of Romanian gastroenterologists, external validity may be constrained by regional differences in training pathways, quality benchmarks, epidemiology, and technology. In light of these differences, we plan to conduct a similar multi-center study across Europe in the near future to test the validity of our findings.

Because the synthetic images are already indistinguishable from reality for most observers, they represent a viable way of enlarging training datasets—practical in the formation of new generations of doctors. Nevertheless, their use in AI development and clinical workflows must be accompanied by automated origin checks or watermarking to prevent accidental dataset contamination and satisfy emerging regulatory requirements. Future work should then integrate authenticity detectors into the synthetic data pipeline and evaluate whether the resulting augmented models translate into measurable reductions in adenoma-miss rates.

## 5. Conclusions

This study demonstrates that gastroenterologists exhibit only moderate accuracy (61.2%, 95% CI: 57.7–64.6%) when distinguishing real, augmented, and synthetic colonoscopy images. Accuracy did not differ significantly by training level, suggesting that additional clinical experience does not notably enhance the ability to detect synthetic images. Detection varied markedly by generation method: CycleGAN images, characterized by noticeable artifacts, were easily identified as synthetic, whereas diffusion-generated images—more anatomically realistic—proved challenging for clinicians to recognize. Inter-rater agreement was fair (κ = 0.30, 95% CI: 0.15–0.43), reflecting widespread uncertainty among observers. Taken together, these findings emphasize that human perception alone is insufficient for reliably identifying synthetic endoscopic images, particularly those produced by advanced diffusion techniques. Objective verification methods should, therefore, complement clinician judgment whenever synthetic data are used, and future studies should test whether such provenance-controlled augmentation translates into lower adenoma-miss rates.

## Figures and Tables

**Table 1:** Cross-tabulation of accuracy by training level.

Subgroup Ratings	Correct	Incorrect
Residents (n = 432)	269	163
Seniors (n = 336)	201	135

**Table 2:** Class-specific performance.

True Class	Sensitivity (%)	Precision (%)
Real	70.7	62.2
Augmented	51.6	58.9
Synthetic	61.3	62.1

**Table 3:** Overall confusion matrix.

Actual vs. Predicted	Real	Augmented	Synthetic
Real	181 (70.7%, 64.9–75.9)	42 (16.4%, 12.4–21.4)	33 (12.9%, 9.3–17.6)
Augmented	61 (23.8%, 19.0–29.4)	132 (51.6%, 45.5–57.6)	63 (24.6%, 19.7–30.2)
Synthetic	49 (19.1%, 14.8–24.4)	50 (19.5%, 15.1–24.8)	157 (61.3%, 55.2–67.1)

**Table 4:** Resident doctors confusion matrix.

Actual vs. Predicted	Real	Augmented	Synthetic
Real	105 (72.9%, 64.9–79.6)	21 (14.6%, 9.7–21.4)	18 (12.5%, 7.9–19.0)
Augmented	31 (21.5%, 15.3–29.3)	78 (54.2%, 46.0–62.2)	35 (24.3%, 17.7–32.5)
Synthetic	30 (20.8%, 15.1–27.9)	28 (19.4%, 13.9–26.4)	86 (59.7%, 51.4–67.6)

**Table 5:** Senior doctors confusion matrix.

Actual vs. Predicted	Real	Augmented	Synthetic
Real	76 (67.9%, 58.5–76.0)	21 (18.8%, 12.5–27.2)	15 (13.4%, 8.1–21.2)
Augmented	30 (26.8%, 19.2–35.9)	54 (48.2%, 39.2–57.3)	28 (25.0%, 18.0–33.7)
Synthetic	19 (17.0%, 11.0–25.4)	22 (19.6%, 13.2–28.3)	71 (63.4%, 54.1–71.8)

**Table 6:** Detection rates per synthesis type.

Generation Type	Correct/Total	Detection Rate	95% CI
CycleGAN	128	100%	97.1–100%
Diffusion-based	29	22.7%	16.3–30.6%

**Table 7:** Prediction patterns within each class by training level.

True Class	χ^2^ (df = 2)	*p*-Value
Real	0.93	0.63
Augmented	1.18	0.56
Synthetic	0.63	0.73

**Table 8:** Breakdown of real image classification by training level.

Training Level	Predicted Real	Predicted Augmented	Predicted Synthetic
Residents	105 (72.9%, 64.9–79.6)	21 (14.6%, 9.7–21.4)	18 (12.5%, 7.9–19.0)
Seniors	76 (67.9%, 58.5–76.0)	21 (18.8%, 12.5–27.2)	15 (13.4%, 8.1–21.2)

**Table 9:** Breakdown of pseudosynthetic image classification by training level.

Training Level	Predicted Real	Predicted Augmented	Predicted Synthetic
Residents	31 (21.5%, 15.3–29.3)	78 (54.2%, 46.0–62.2)	35 (24.3%, 17.7–32.5)
Seniors	30 (26.8%, 19.2–35.9)	54 (48.2%, 39.2–57.3)	28 (25.0%, 18.0–33.7)

**Table 10:** Breakdown of synthetic image classification by training level.

Training Level	Predicted Real	Predicted Augmented	Predicted Synthetic
Residents	30 (20.8%, 15.1–27.9)	28 (19.4%, 13.9–26.4)	86 (59.7%, 51.4–67.6)
Seniors	19 (17.0%, 11.0–25.4)	22 (19.6%, 13.2–28.3)	71 (63.4%, 54.1–71.8)

## Data Availability

The data presented in this study are available on request from the corresponding author due to privacy reasons.
